# Longitudinal Structural and Functional Evaluation of Dark-without-Pressure Fundus Lesions in Patients with Autoimmune Diseases

**DOI:** 10.3390/diagnostics14202289

**Published:** 2024-10-15

**Authors:** Marco Lombardo, Federico Ricci, Andrea Cusumano, Benedetto Falsini, Carlo Nucci, Massimo Cesareo

**Affiliations:** 1 Department of Experimental Medicine, Ophthalmology Unit, University of Rome Tor Vergata, 00133 Rome, Italy; 2 Macula & Genoma Foundation, 00133 Rome, Italy

**Keywords:** dark without pressure, autoimmune diseases, optical coherence tomography, OCT, fluorescein angiography, indocyanine green angiography

## Abstract

Objectives: The main objective of this study was to report and investigate the characteristics and longitudinal changes in dark-without-pressure (DWP) fundus lesions in patients with autoimmune diseases using multimodal imaging techniques. Methods: In this retrospective observational case series, five patients affected by ocular and systemic autoimmune disorders and DWP were examined. DWP was assessed by multimodal imaging, including color fundus photography (CFP), near-infrared reflectance (NIR), blue reflectance (BR), blue autofluorescence (BAF), optical coherence tomography (OCT), OCT-angiography (OCT-A), fluorescein angiography (FA) and indocyanine green angiography (ICGA), and functional testing, including standard automated perimetry (SAP) and electroretinography (ERG). Follow-up examinations were performed for four out of five patients (range: 6 months–7 years). Results: DWP fundus lesions were found in the retinal mid-periphery and were characterized by the hypo-reflectivity of the ellipsoid zone on OCT. DWP appeared hypo-reflective in NIR, BR and BAF, and exhibited hypo-fluorescence in FA in two patients while showing no signs in one patient. ICGA showed hypo-fluorescent margins in one patient. SAP and ERG testing did not show alterations attributable to the DWP lesion. Follow-up examinations documented rapid dimensional changes in DWP even in the short term (1 month). Conclusions: This study suggests a possible association between autoimmune diseases and DWP. New FA and ICGA features were described. The proposed pathogenesis hypotheses may operate as a basis for further investigation of a lesion that is still largely unknown. Large population studies would be necessary to confirm whether there is a higher incidence of DWP in this patient category.

## 1. Introduction

Dark-without-pressure (DWP) fundus lesions are rare asymptomatic retinal findings first named in 1975 [1]. Despite advances in diagnostic and imaging techniques, the pathogenesis of these lesions remains unknown. DWP has been described in various systemic and ocular diseases such as hemoglobinopathy [1,2], congenital hypertrophy of the retinal pigment epithelium [3,4,5], Ebola virus disease [6] and toxoplasmosis [7,8], but also in otherwise asymptomatic patients [9].

This work aimed to describe a case series of patients with simultaneous DWP and autoimmune diseases. Additionally, we suggested novel pathogenesis hypotheses based on our results and the latest evidence in the scientific literature.

## 2. Materials and Methods

We identified a case series of five patients, afferent to the ophthalmology department of the University of Tor Vergata in Rome, in whom, during complete ophthalmological examinations, a dark area in the mid-peripheral retina, consistent with the diagnosis of DWP, was accidentally detected. All patients underwent multimodality imaging, including color fundus photography (CFP) with a Digital Non-Mydriatic Retinal Camera (Canon CR-2), near-infrared reflectance (NIR), blue reflectance (BR), blue autofluorescence (BAF) and spectral-domain optical coherence tomography (OCT) with Spectralis (Heidelberg Engineering, Heidelberg, Germany). In three out of five patients, the DWP location permitted the execution of OCT-angiography (OCT-A) using RTVue XR Avanti with AngioVue (Optovue, Inc., Fremont, CA, USA). Two patients underwent fluorescein angiography (FA) and indocyanine green angiography (ICGA), and one patient FA only. Visual field examinations with standard automated perimetry (SAP) with a Humphrey Field Analyzer (30-2 and 60-4 test patterns) were performed in all patients, and standard electroretinography (ERG) with Retimax (CSO, Florence, Italy) according to the International Society for Clinical Electrophysiology of Vision (ISCEV) protocol was performed in two patients. In four patients, it was possible to carry out follow-up examinations over time through multimodal imaging.

The study was approved by the Internal Review Board of the University of Tor Vergata (ethics approval ID: 263.22). The described research adhered to the tenets of the Declaration of Helsinki. Written informed consent was obtained from all subjects.

## 3. Results

Table 1 summarizes the demographic, clinical, functional and structural characteristics of the patients included in the present study.

CASE 1. A 27-year-old Caucasian female was followed at our neuro-ophthalmology clinic to monitor the outcomes of idiopathic intracranial hypertension with bilateral papilledema. The patient suffered from systemic lupus erythematosus and has been treated with hydroxychloroquine and oral prednisone for 8 years. The patient was asymptomatic. The best corrected visual acuity (BCVA) was 0.2 logMAR (20/32) in the right eye (RE) and 0.0 logMAR (20/20) in the left eye (LE) with a spherical equivalent of −0.50 and −0.25 diopters, respectively. The anterior segment was within normal limits and no signs of ocular inflammation were found. Fundus examination revealed a pale optic disc in both eyes and a crescent-shaped dark spot of approximately 5 papillary diameters in the superior nasal mid-periphery of the LE. An OCT scan at the lesion level showed the attenuation of the reflectivity of the ellipsoid zone (EZ) band without the involvement of the other layers. The findings were consistent with the diagnosis of DWP. The 60-4 and 30-2 SITA-SAP showed scotomas compatible with the optic atrophy, but no scotoma attributable to the DWP lesion was found.

The patient missed subsequent follow-up appointments and returned to our Center after 7 years without reporting changes in visual function (BCVA and SAP). The previously present DWP lesion was not detectable either at ophthalmoscopy or at multimodal imaging (NIR, BR, BAF, CFP, OCT, OCT-A). FA and ICGA were not performed, as the patient was asymptomatic.

**Figure 1:**
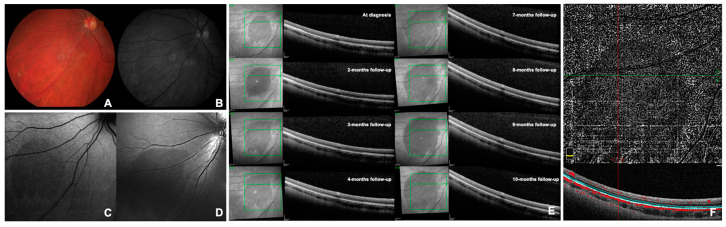
Selected images of dark without pressure from Case 2 (see text). Panel (**A**) shows the color retinography during the 3-month follow-up examination. Panel (**B**) shows the red-free retinography during the 3-month follow-up examination. Panel (**C**) shows the blue autofluorescence at the time of diagnosis. Panel (**D**) shows the blue reflectance during the 3-month follow-up examination. Panel (**E**) shows infrared optical coherence tomography at the time of diagnosis and the subsequent follow-ups. Panel (**F**) shows the optical coherence tomography–angiography with the flow limits set at the ellipsoid zone level at the time of diagnosis.

CASE 2. A 23-year-old Caucasian female was followed as an outpatient for recurrent anterior uveitis predominantly affecting the RE. The patient was affected by juvenile idiopathic arthritis in therapy with methotrexate, certolizumab pegol and topical dexamethasone in the RE. BCVA was 0.0 logMAR (20/20) in both eyes (spherical equivalent: −4.00 diopters in RE and −4.25 diopters in LE). A 0.5+ grade of anterior chamber cells (SUN grading) was noted in the RE with no other clinical signs of uveitis. The anterior segment examination of the fellow eye was unremarkable. The fundus examination of both eyes appeared within normal limits except for an oval-shaped dark spot of approximately 4 papillary diameters in the inferior nasal mid-periphery of the LE diagnosed as DWP (Figure 1A). The lesion was more evident by red-free light ophthalmoscopy and was hypo-fluorescent on BAF and hypo-reflective at NIR and BR examinations (Figure 1B–D). At the OCT scans, the dark area corresponded to an attenuation of the EZ (Figure 1E). At OCT-A, the en-face examination of the outer retina showed a decrease in the projection artifacts usually present in this normally avascular slab when using this device. This effect became even more evident by manually setting the upper and lower limits of flow detection, including only the EZ band (Figure 1F). The superficial and deep capillary plexus and the choriocapillaris did not appear to be affected. The visual field examinations with 60-4 and 30-2 SITA-SAP were within normal limits. The scotopic ERG of the RE was “slightly” reduced in amplitude, whereas all ERG findings of the LE were within normal limits. At follow-up visits, we documented a constant monthly increase in lesion diameter by OCT (Figure 1E) in the absence of visual symptoms. FA showed the presence of late papillary leakage with peripheral vasculitis in the RE and only mild generalized blood–retinal barrier breakdown in the LE. The DWP lesion was silent both at the FA and ICGA; the latter exam was unremarkable in both eyes throughout the entire examination.

**Figure 2:**
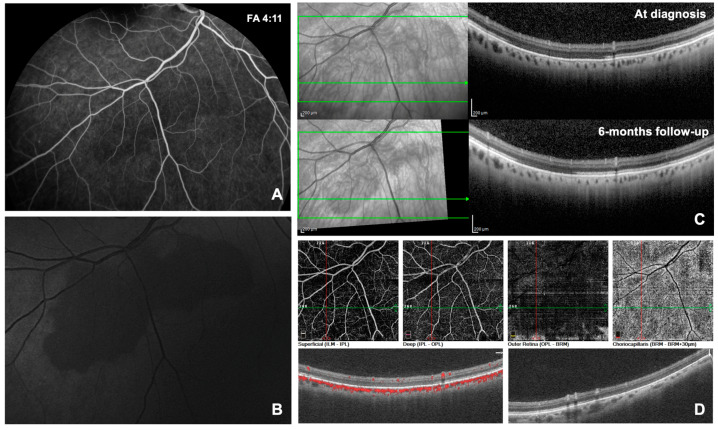
Selected images of dark without pressure from Case 3 (see text). Panel (**A**) shows the mid-phase fluorescein angiography at the time of diagnosis. Panel (**B**) shows the blue reflectance at the time of the diagnosis. Panel (**C**) shows the infrared optical coherence tomography at the diagnosis and during the follow-up examination after 6 months. Panel (**D**) shows the optical coherence tomography angiography at the time of the diagnosis.

CASE 3. A 48-year-old Caucasian male presented to our Retina Center to perform FA to investigate optic neuropathy with a recent onset of microcystic macular edema in both eyes. He assumed ramipril for systemic hypertension and no drugs potentially toxic to the retina. The patient presented a BCVA of 0.1 logMAR (20/25) in the RE and hand motion in the LE. The patient was pseudophakic and a PreserFlo micro-shunt was present with effective filtrating blebs in both eyes. He assumed topical timolol, brimonidine, brinzolamide, and bimatoprost in both eyes for advanced glaucomatous optic neuropathy. The intraocular pressure was 9 mmHg in the RE and 10 mmHg in the LE. The patient had no clinical signs of intraocular inflammation. FA showed hypo-fluorescence of the optic disc in both eyes, perivascular leakage in the temporal periphery of the LE, and mild late-phase pooling in the macular area in both eyes. We related the microcystic macular edema to the advanced optic neuropathy. The patient did not present signs of epitheliopathy. A trapezoid-shaped area of approximately 4 papillary diameters, that remained hypo-fluorescent in all phases of the angiography, was highlighted in the inferior mid-periphery of the RE (Figure 2A). This area was barely visible in ophthalmoscopy as a dark spot but was better identifiable through red-free light; it was hypo-fluorescent on BAF and hypo-reflective at NIR and BR (Figure 2B). The lesion, diagnosed as DWP, corresponded to an attenuation of the reflectivity of the EZ band at the OCT scans (Figure 2C). OCT-A showed similar findings to Case 2 (Figure 2D). Brain magnetic resonance imaging showed signs of multifocal leukoencephalopathy of probable chronic microvascular nature. SITA-SAP revealed absolute and relative peripheral scotomas with a partial sparing of the central region in the RE, while the central area in the LE was involved. Visual field examinations did not allow for highlighting any signs attributable to the DWP spot. The patient has been diagnosed with microscopic polyangiitis associated with anti-neutrophil cytoplasmic antibody (p-ANCA). At the 6-month follow-up visit, the DWP lesion had decreased in size; the patient had started treatment with prednisone and methotrexate two months before.

CASE 4. A 53-year-old African male was examined at our low vision Center for the first time. The patient suffered from type 2 diabetes, moderate renal insufficiency, interstitial lung disease and dyslipidemia, had a history of acute autoimmune polyneuropathy with positive anti-myelin-associated-glycoprotein antibody and a positive Treponema Pallidum hemagglutination assay test. The patient’s systemic therapy included insulin, lansoprazole, atorvastatin, pregabalin, tamsulosin and cholecalciferol. The BCVA was 1.6 logMAR (20/796) in both eyes. The patient presented an incipient cataract, no signs of ocular inflammation and optic disc atrophy in both eyes. Fundus examination of the temporal mid-periphery of the RE revealed an oval-shaped DWP spot of approximately 3 papillary diameters. It appeared hypo-reflective at NIR and corresponded to an attenuation of the EZ band reflectivity at the OCT scans. FA and ICGA examinations were contraindicated due to the patient’s general health condition. The perimetry tests resulted in a high number of errors but showed absolute and relative scotomas involving the entire visual field in both eyes, consistent with the patient’s optic atrophy; it was not possible to highlight any signs attributable to the DWP lesion.

**Figure 3:**
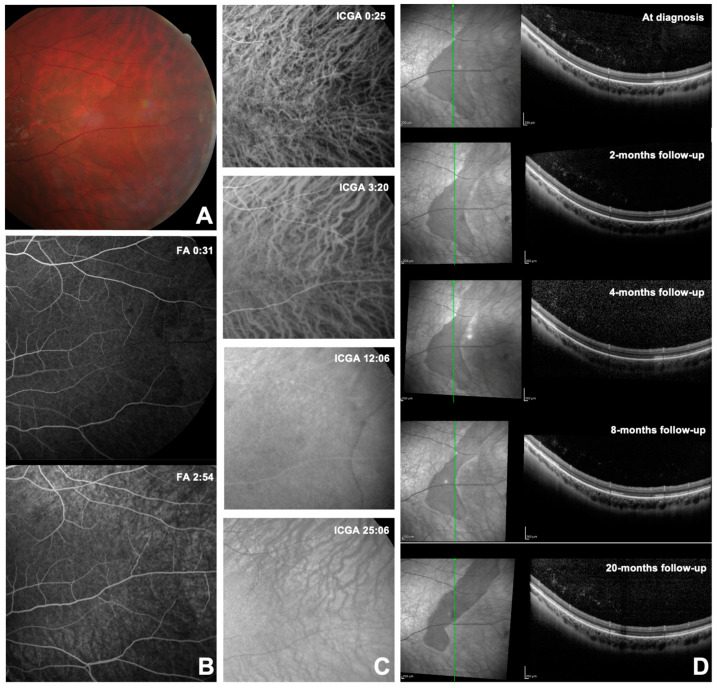
Selected images of dark without pressure from Case 5 (see text). Panel (**A**) shows color retinography at the time of the diagnosis. Panel (**B**) shows the early and mid-phases of fluorescein angiography at the time of diagnosis. Panel (**C**) shows the indocyanine green angiography phases at the time of the diagnosis. Panel (**D**) shows the infrared optical coherence tomography at the time of the diagnosis and the subsequent follow-ups.

CASE 5. A 30-year-old Caucasian male presented to our Retina Center for a follow-up examination of an extrafoveal inactive fibrotic inflammatory macular neovascularization in the RE, previously treated by five aflibercept intravitreal injections. The patient was diagnosed with primary sclerosing cholangitis and supplemented with ursodeoxycholic acid. The patient was asymptomatic; his BCVA was 0.0 logMAR (20/20) in both eyes (spherical equivalent: −0.50 diopters in the RE and −1.00 diopters in the LE). The anterior segment of both eyes was within normal limits, and no signs of ocular inflammation were found. Fundus examination showed no changes in the RE but a crescent-shaped DWP area in the temporal mid-periphery of the LE adjacent to an area of white without pressure was detected. The lesion, measuring approximately 4 papillary diameters, had a well-defined inferior margin, while the superior margin was not (Figure 3A). DWP was slightly hypo-fluorescent on BAF while well-defined through BR and NIR retinography. The FA revealed a delay in perfusion of the vessels feeding the affected area, and the DWP lesion showed a faint mask hypo-fluorescence all along the exam duration (Figure 3B). On ICGA, the spot remained not visible except for the temporal hypo-fluorescent margins in the late-intermediate phase (Figure 3C). OCT examination showed that the hypo-reflective area corresponded to an attenuation of the reflectivity of the EZ band (Figure 3D). The OCT-A was of poor quality due to the lesion location but demonstrated findings like Cases 2 and 3. The 30-2 SITA-SAP showed paracentral relative scotomas in the RE attributable to previous macular neovascularization, while the LE was within normal limits. The 60-4 SAP was unremarkable in both eyes and the standard ERG was within normal limits in both eyes. The DWP lesion demonstrated a progressive reduction in size over time, as demonstrated by follow-up OCT scans performed at 2, 4 and 8 months. The size reduction of the lesion at NIR was correlated to the gradual restoration of EZ band reflectivity on OCT (Figure 3D). After 20 months, the DWP lesion appeared significantly reduced in extension in the lower part, but the superior and temporal margins had widened (Figure 3D). The patient reported having a flu-like episode 2 weeks earlier with no ocular symptoms.

## 4. Discussion

We presented a case series of five patients affected by autoimmune diseases in whom a DWP fundus lesion was identified in the retinal mid-periphery.

The appearance of DWP in IR, BR, BAF, OCT and OCT-A, as well as the evolution of the lesions over time, showed findings compatible with the available scientific literature. Similarly, visual field examinations and ERG did not show signs attributable to the lesions. However, FA and ICGA showed findings never previously reported.

The presence of DWP as a comorbidity of autoimmune diseases may suggest a different pathophysiology of these lesions.

Our hypothesis is as follows: in predisposed patients with a dysregulated immune system, or after retinal damage caused by an infection [7], an immune response involving the outer retina can be triggered. The target would be an antigen, not yet well-characterized, present at the mitochondrial level of the EZ. This reaction temporarily alters the reflectivity of this normally hyper-reflective OCT band, making it hypo-reflective. The antigen could be present at the level of an active transport enzyme of the mitochondrial membrane, of which the dysfunction could lead to a reversible alteration of the relative molecular concentrations of substances affecting the reflectivity of this band.

This theory could explain the progressive and complete restoration of the EZ band without photoreceptor loss as a return to normal relative molecular status. If these hypotheses were verified, the presence of DWP could be an indication of the poor control of the underlying autoimmune or infective disease, and its resolution could instead represent the return to the immune system’s equilibrium. The same abnormal immune response against self-antigens could also be caused by a process of molecular mimicry after an infection or a vaccine. However, it is difficult to establish a temporal cause–effect relationship due to the lack of symptoms.

A similar pathophysiological mechanism has been proposed for multiple evanescent white dot syndrome (MEWDS), but the clinical and multimodal imaging features are different. We believe that the dissimilar appearances may be explained by the theory that MEWDS is a “choriocapillaritis”, which may contribute to the ICGA hypo-fluorescence along with a deficient dye uptake by a damaged RPE. The late hypo-fluorescence of spots in ICGA is found in MEWDS but not in primary “photoreceptoritis”, a group of which DWP could be a part [10].

The appearance of the lesion in FA differed in Cases 3 and 5 from Case 2 and previously reported cases where the lesion was not visible [1,4,11]. A recent publication reported an even more dissimilar aspect of DWP in FA [12]. The different FA presentations may be due to the dynamic nature of the exam, which may reflect the same dynamic nature of the DWP lesion and may have been performed at different pathophysiological moments.

The fact that ICGA was mostly unremarkable suggests that DWP does not have a primarily vascular origin and that the choriocapillaris is not involved. Occasionally, we observed hypo-fluorescent margins of the lesion in the late-intermediate phase in Case 5, but we are not able to provide a clear explanation for this novel finding.

A recent study showed that the vascular density of the deep vascular complex (DVC) was reduced in areas of DWP compared to healthy contralateral areas [13].

It has been shown that the DVC and not just the choroidal circulation is important for the sustenance of the photoreceptors [14]; otherwise, the retinal circulation is known to have intrinsic self-regulation based on the needs of its cells. The decrease in vessel density in this retinal plexus may be due to a lower metabolic demand on the photoreceptors, given the putative involvement of the mitochondria of the EZ.

It must be considered that the nature of the band called EZ is still a topic of debate [15]. Therefore, our hypotheses may have to be revised in case of future discoveries.

As previously reported [3], ERG results suggest that the possible rod dysfunction in these patients is not generalized. It may eventually be limited to the DWP areas that are too small to affect this type of full-field examination. Similar conclusions can be drawn from the SAP findings.

Similarly to previous research findings, we did not find any associations with the vitreoretinal interface in our patients. DWP may appear more evident in axial myopic eyes because of better visibility in a thinner retina; on the other hand, a longer eye could have a more vulnerable outer retina due to a thinner choroid and choriocapillaris.

## 5. Conclusions

We described a series of patients suffering from autoimmune diseases with the contemporary presence of ocular diseases and DWP. They underwent functional and structural examinations, which allowed us to formulate pathogenetic hypotheses on this interesting lesion, which is still not described enough in the literature. To the best of our knowledge, we reported an aspect of DWP that has not yet been described in FA and ICGA. Our suggestion is that there may be an association between autoimmune diseases and DWP, but large-scale case–control studies will be needed to verify these hypotheses.

To date, we suppose DWP not to be a distinct disease but a clinical sign that can be present in normal and pathological conditions (autoimmune, infectious or other). It should not raise concerns but could be a marker of underlying disease activity.

The limitations of this study include the retrospective nature, the absence of a control group and the limited number of patients. Systemic blood parameters related to the underlying autoimmune disease control were not evaluated for all patients, and the frequency of follow-up and the execution of different functional and structural exams were not the same for each patient.

We believe that the increasing use of ultra-widefield retinal imaging methods, together with adaptive optics retinal images, may provide further answers and a better characterization of this retinal finding.

## Figures and Tables

**Table 1:** Demographic, clinical, functional and structural characteristics of the patients included.

Case Number	1	2	3	4	5
Age and Gender	27-year-old female	23-year-old female	48-year-old male	53-year-old male	30-year-old male
Ethnicity	Caucasian	Caucasian	Caucasian	African	Caucasian
Autoimmune disease	Systemic lupus erythematosus	Juvenile idiopathic arthritis	p-ANCA-associated microscopic polyangiitis	Anti-MAG autoimmune polyneuropathy	Primary sclerosing cholangitis
Ocular disease (eye without DWP)	Atrophic outcomes of papilledema	Recurrent anterior uveitis	Vascular and glaucomatous optic neuropathy	Optic atrophy of uncertain origin	Fibrotic inflammatory macular neovascularization
Ocular disease (eye with DWP)	Atrophic outcomes of papilledema	Recurrent anterior uveitis	Vascular and glaucomatous optic neuropathy	Optic atrophy of uncertain origin	Within normal limits
Location of DWP	Superior nasal mid-periphery	Inferior-nasal mid-periphery	Inferior mid-periphery	Temporal mid-periphery	Temporal mid-periphery
Ophthalmoscopic aspect of DWP	Crescent-shaped dark spot with defined margins (5 papillary diameters)	Oval-shaped dark spot with defined margins (4 papillary diameters)	Trapezoid-shaped dark spot with defined margins (4 papillary diameters)	Oval-shaped dark spot with defined margins (3 papillary diameters)	Crescent-shaped dark spot with indefinite superior margins (4 papillary diameters)
NIR	Hypo-reflective	Hypo-reflective	Hypo-reflective	Hypo-reflective	Hypo-reflective
BR	Hypo-reflective	Hypo-reflective	Hypo-reflective	Hypo-reflective	Hypo-reflective
BAF	Hypo-fluorescent	Hypo-fluorescent	Hypo-fluorescent	Hypo-fluorescent	Hypo-fluorescent
OCT	Attenuation of the reflectivity of the EZ band	Attenuation of the reflectivity of the EZ band	Attenuation of the reflectivity of the EZ band	Attenuation of the reflectivity of the EZ band	Attenuation of the reflectivity of the EZ band
OCTA	Not available	SCP, DCP, CC within normal limits; decreased projection artifacts in the outer retina	SCP, DCP, CC within normal limits; decreased projection artifacts in the outer retina	Not available	SCP, DCP, CC within normal limits; decreased projection artifacts in the outer retina
FA	Not available	Not visible	Hypo-fluorescence in all phases	Not available	Hypo-fluorescence in all phases
ICGA	Not available	Not visible	Not available	Not available	Hypo-fluorescence margins in the late-intermediate phase
SAP (30-2; 60-4)	Abnormal, no apparent defects attributable to DWP	Within normal limits	Abnormal, no apparent defects attributable to DWP	Abnormal, no apparent defects attributable to DWP	Within normal limits
ERG	Not available	Reduced scotopic amplitude in the fellow eye	Not available	Not available	Within normal limits
Longitudinal evaluation	Disappeared at the 7-year follow-up	Concentric increase in size over 10 months	The size decreased at the 6-month follow-up	Not available	The size decreased over 8 months and then increased at the 20-month follow-up

Abbreviations: BCVA, best-corrected visual acuity; DWP, dark without pressure; NIR, near-infrared reflectance; BR, blue reflectance; BAF, blue autofluorescence; OCT, optical coherence tomography; OCT-A, optical coherence tomography–angiography; SCP, superficial capillary plexus; DCP, deep capillary plexus; CC, choriocapillaris; FA, fluorescein angiography; ICGA, indocyanine green angiography; SAP, standard automated perimetry; ERG, electroretinography.

## Data Availability

The datasets generated during the current study are available from the corresponding author upon reasonable request.
